# The Tumour Suppressor CYLD Is Required for Clathrin-Mediated Endocytosis of EGFR and Cetuximab-Induced Apoptosis in Head and Neck Squamous Cell Carcinoma

**DOI:** 10.3390/cancers14010173

**Published:** 2021-12-30

**Authors:** Rin Liu, Satoru Shinriki, Manabu Maeshiro, Mayumi Hirayama, Hirofumi Jono, Ryoji Yoshida, Hideki Nakayama, Hirotaka Matsui

**Affiliations:** 1 Department of Molecular Laboratory Medicine, Graduate School of Medical Sciences, Kumamoto University, Kumamoto 860-8556, Japan; r-liu@kuh.kumamoto-u.ac.jp (R.L.); mayumihi@kuh.kumamoto-u.ac.jp (M.H.); 2 Department of Oral and Maxillofacial Surgery, Faculty of Life Sciences, Kumamoto University, Kumamoto 860-8556, Japan; kitakyushu@kuh.kumamoto-u.ac.jp (M.M.); fcryoshida1126@kuh.kumamoto-u.ac.jp (R.Y.); hinakaya@kumamoto-u.ac.jp (H.N.); 3 Department of Clinical Pharmaceutical Sciences, Graduate School of Pharmaceutical Sciences, Kumamoto University, Kumamoto 860-8556, Japan; hjono@kuh.kumamoto-u.ac.jp

**Keywords:** CYLD, EGFR, cetuximab, clathrin-mediated endocytosis, head and neck squamous cell carcinoma

## Abstract

**Simple Summary:**

Epidermal growth factor receptor (EGFR) is a target for the therapeutic antibody cetuximab (CTX) in head and neck squamous cell carcinoma (HNSCC). Identification of its predictive biomarkers and potentiation of CTX-based therapies are important. In this study, we found that the N-terminal portion of cylindromatosis (CYLD) was required for clathrin-mediated endocytosis (CME) and degradation of EGFR. Loss of CYLD limited EGFR to lipid rafts and inhibited CTX-induced apoptosis. Destruction of lipid rafts by cholesterol removers restored EGFR CME and CTX activity in CYLD-downregulated cells. Our findings provide novel insights into the molecular mechanisms underlying EGFR trafficking and resistance to CTX, and suggest the usefulness of CTX-based therapy combined with cholesterol-lowering drugs in HNSCC.

**Abstract:**

Epidermal growth factor receptor (EGFR) is frequently overexpressed in head and neck squamous cell carcinoma (HNSCC) and is a target for the therapeutic antibody cetuximab (CTX). However, because only some patients have a significant clinical response to CTX, identification of its predictive biomarkers and potentiation of CTX-based therapies are important. We have recently reported a frequent downregulation of cylindromatosis (CYLD) in primary HNSCC, which led to increased cell invasion and cisplatin resistance. Here, we show that CYLD located mainly in lipid rafts was required for clathrin-mediated endocytosis (CME) and degradation of the EGFR induced by EGF and CTX in HNSCC cells. The N-terminus containing the first cytoskeleton-associated protein-glycine domain of CYLD was responsible for this regulation. Loss of CYLD restricted EGFR to lipid rafts, which suppressed CTX-induced apoptosis without impeding CTX’s inhibitory activity against downstream signalling pathways. Disruption of the lipid rafts with cholesterol-removing agents overcame this resistance by restoring CME and the degradation of EGFR. Regulation of EGFR trafficking by CYLD is thus critical for the antitumour activity of CTX. Our findings suggest the usefulness of a combination of cholesterol-lowering drugs with anti-EGFR antibody therapy in HNSCC.

## 1. Introduction

Head and neck squamous cell carcinoma (HNSCC), a squamous cell carcinoma, is the sixth most common type of cancer worldwide. Squamous cell carcinoma is a tumour characterized by an abnormal and quick growth of keratinocytes in the epidermis. Although this kind of tumour is usually associated with ultraviolet light exposure and tobacco and alcohol use, HNSCC may be more linked to previous human papillomavirus (HPV) infection in up to 25% of cases [1]. About two-thirds of patients with HNSCC have advanced disease at diagnosis [2]. Therapeutic options remain limited in patients with recurrent or metastatic HNSCC not expressing programmed cell death ligand 1 (PD-L1) or who have contraindications to anti-programmed cell death protein 1 (PD-1) inhibitor treatment [3]. Limited biomarkers such as those positive for HPV or p16(INK4a) potentially predict the radiosensitivity of HNSCC to a certain extent, but these are not still sufficient [4]. A better understanding of the biological mechanisms responsible for treatment efficacy and resistance is needed to improve patients’ outcomes via the design of new therapeutic combinations, including consideration of minimally invasive surgery [5].

Epidermal growth factor (EGF) receptor (EGFR) is a receptor tyrosine kinase that serves as a master control for cell growth and differentiation pathways in HNSCC [6]. EGFR is frequently overexpressed (~90%) or the gene is amplified in primary HNSCC, which is thought to correlate with carcinogenesis, metastasis, and poor prognosis [7]. Cetuximab (CTX, ICM-225) is a human/murine chimeric IgG1 monoclonal antibody used to treat HNSCC. CTX binds structurally to Domain III of EGFR’s tethered extracellular domain, thereby competitively interfering with ligand binding at Domains I and III and stabilizing the receptor in the closed conformation [8,9]. The favourable clinical efficacy of CTX was demonstrated by administering this antibody together with platinum-based agents in recurrent or metastatic HNSCC, and using it as a radiosensitizer as a component of definitive radiotherapy for locally advanced cases and cisplatin-unfit patients [10,11]. Because of these findings, CTX has become a standard therapeutic agent for HNSCC treatment. However, individual HNSCC patients show wide-ranging degrees of response to CTX, with only a few patients demonstrating significant tumour shrinkage [12]. Primary resistance to CTX is mainly due to aberrations in the KRAS, NRAS, and EGFR genes [13], which are often found in other cancer backgrounds, including colorectal cancer (CRC) and non-small cell lung cancer (NSCLC) but are uncommon in HNSCC tumours in CTX-naïve patients [14]. In addition, biomarkers predicting the responsiveness of CTX and oncogenic molecules potentially related to CTX resistance are limited [15]. Moreover, CTX treatment inevitably induces an acquired resistance through diverse mechanisms, even in cases with an effective initial response [16,17]. At present, reliable predictive biomarkers of the clinical activity of CTX, either alone or combined with chemotherapy or radiotherapy, are lacking in the HNSCC setting, so identification of such biomarkers and potentiation of CTX-based therapies by characterizing the molecular mechanisms involved in CTX therapeutic activity, adverse events, and acquired resistance are important [12,16,18].

The molecular mechanisms underlying EGFR activation and intracellular trafficking have been documented in detail [19]. A conformational change in the extracellular domain of the receptor during ligand binding allows ligand-mediated EGFR activation. Dimerization is critical for ligand-stimulated EGFR autophosphorylation, activation, and internalization [20]. Phosphotyrosine-binding proteins are thus engaged, so many signal transduction pathways, including the Ras-Raf-MEK-ERK, PI3K-AKT, and JAK-STAT3 cascades, are activated. These pathways are involved in the carcinogenesis and invasiveness of many cancer types [21]. Ligand-induced receptor internalization requires receptor tyrosine kinase activity for entry into clathrin-coated pits. After endocytosis, ligand–receptor complexes are directed mainly to the lysosomes but can also be recycled to cell surfaces. EGF also causes intracellular trafficking of a small fraction of the receptor to the endoplasmic reticulum (ER) and nucleus [22,23]. Modulations in not only the downstream pathways but also EGFR trafficking and function can result in oncogenesis or change the outcomes of antineoplastic therapies [16,19].

In addition to ligands, CTX is internalized as an antibody–receptor complex with EGFR, even though the antibody prevents EGFR stimulation by ligands. In contrast to stimulation by ligands, antibody-dependent EGFR internalization does not require receptor kinase activity [24]. Although the route has not been clarified, the internalized antibody–receptor complex can be degraded in lysosomes [24], recycled to cell surfaces [24,25], or sorted to nuclei through the ER [25]. EGFR downregulation after CTX treatment has been shown to predict antitumour effects in CRC [26]. However, little is still known about antibody-induced EGFR trafficking, the factors commonly involved in ligand- and antibody-induced receptor trafficking, and the clinical significance of receptor trafficking. Elucidating the molecular events that affect EGFR trafficking during tumour progression may contribute to understanding the mechanisms regulating tumour progression and to improving CTX efficacy by overcoming primary resistance.

The cylindromatosis gene (CYLD) was first found to be associated with familial cylindromatosis, a condition involving multiple skin tumours [27]. CYLD protein functions as a deubiquitinase through the ubiquitin-specific protease (USP) domain, which mainly removes lysine 63 (K63)-linked polyubiquitin chains on the target proteins [28,29] and thereby regulates various signalling pathways, including nuclear factor-κB (NF-κB), Wnt/β-catenin, c-Jun N-terminal kinase, p38 mitogen-activated protein kinase, and Hippo and Notch [29,30]. CYLD also has three cytoskeleton-associated protein-glycine (CAP-Gly; CG) domains. CYLD binds directly and indirectly to tubulin and microtubules via CG domains to regulate microtubule dynamics, with certain differences in tubulin-binding affinity and interacting partners among these CG domains [29]. Accumulating evidence has established the tumour-suppressive role of CYLD [30]. Loss-of-function mutations in this gene and reduced expression are found in various cancer types [31,32,33]. We recently reported that although alterations in CYLD were rare, CYLD protein expression was often reduced, particularly in invasive lesions in primary HNSCC, which was associated with poor prognosis [34]. We found that loss of CYLD promoted epithelial–mesenchymal transition-like changes and cell invasion by activating transforming growth factor-β signalling. Despite altered CYLD expression having been observed in various cancers in which EGFR signalling contributed to cancer development and progression, the roles of CYLD in EGFR signalling and response to anti-EGFR therapies remain unknown. Limited reports have suggested the involvement of CYLD in the spatial regulation of EGFR signals by showing the requirement for CYLD in the formation of dorsal ruffles in focal adhesion mediated by EGFR and integrin [35]. As a notable result, we showed that CYLD protein downregulation led to cisplatin resistance in HNSCC [36], with the assumption that HNSCC cells with a lower CYLD expression have many opportunities to be exposed to CTX-based therapies because of the current treatment strategy for HNSCC.

Therefore, in this study, we investigated how CYLD is associated with EGFR trafficking and signalling, and the antitumour activity of CTX in HNSCC cells. We present evidence that CYLD is essential for clathrin-mediated endocytosis (CME) and degradation of EGFR induced by both EGF and CTX in HNSCC cells. As an important result, the loss of CYLD induced resistance to CTX by inhibiting EGFR’s exiting lipid rafts. This resistance was overcome by disrupting the lipid rafts with cholesterol-removing agents via restoring CME and degrading EGFR. These findings provide new insights into the mechanisms underlying EGFR trafficking and responsiveness to anti-EGFR antibody therapy.

## 2. Materials and Methods

### 2.1. Reagents and Cell Culture

The anti-EGFR monoclonal antibody CTX was purchased from Merck (Tokyo, Japan). The nystatin and methyl-β-cyclodextrin (mβCD) used to reduce cholesterol content, the cholera toxin subunit B (recombinant) Alexa Fluor 594 conjugate used to detect lipid rafts, and cycloheximide (CHX) were obtained from Sigma-Aldrich (St. Louis, MO, USA). The clathrin inhibitor chlorpromazine (CPZ) was obtained from Cayman Chemical (Ann Arbor, MI, USA). The Cell Resource Center for Biomedical Research, Tohoku University (Sendai, Japan) donated the human HNSCC cell lines HSC3 and Ca9-22. The TSU cells were a kind gift from Drs Shuichi Kawashiri and Koroku Kato (Kanazawa University, Ishikawa, Japan). Cells were grown in Dulbecco’s modified Eagle’s medium (Thermo Fisher Scientific, Waltham, MA, USA) supplemented with 10% heat-inactivated foetal bovine serum (Thermo Fisher Scientific) in 5% CO_2_ at 37 °C.

### 2.2. Transfection with siRNA

Cells were transfected with siRNA by using Lipofectamine 2000 (Thermo Fisher Scientific) according to the manufacturer’s protocol. The siCYLD sequences were the following: sense 5′-GAUUGUUACUUCUAUCAAAtt-3′ and antisense 5′-UUUGAUAGAAGUAACAAUCtt-3′. The sequences of siCYLD-UTR, which targets the sequence containing the 3′-UTR region of the CYLD gene, were as follows: sense 5′-GCAGAGUCCUAACGUUGCAtt-3′ and antisense 5′-UGCAACGUUAGGACUCUGCtt-3′.

### 2.3. Construction of Plasmids

The plasmids pDEST-HA-CYLD and pENTR-HA-CYLD-C601A were kind gifts from Stephen Elledge (Addgene plasmids #15506, #60027). The pDEST-HA-CYLD-C601A that expressed the C601A mutant CYLD was made by subcloning the region between the FseI and SnaBI sites of pENTR-HA-CYLD-C601A into the corresponding region in pDEST-HA-CYLD. The CYLD deletion mutants, i.e., ΔCG1, ΔCG1/2, ΔUSP, and CG1, were based on the pDEST-HA-CYLD plasmid by using the KOD-Plus-Mutagenesis Kit (TOYOBO, Tokyo, Japan). Table 1 gives the primers used. Cells were transfected by using Polyethylenimine MAX (Polysciences, Warrington, PA, USA) according to the manufacturer’s instructions.

### 2.4. Cell Viability Assay

Cells (7.0 × 10^4^) were plated in 12-well plates and transfected with siRNAs or plasmids after 24 h of incubation. At 48 h after transfection, CTX (10, 50, 100, 200 µg/mL) was added with serum-free medium. After 72 h, the number of living cells was counted by using trypan blue.

### 2.5. Immunofluorescence

Transfection was performed 24 h after cells were seeded on cover glasses, then, 48 h after transfection, the cells were starved overnight in serum-free medium. After stimulation with EGF (100 ng/mL) or CTX (100 µg/mL), cells were fixed with 4% paraformaldehyde (PFA) for 15 min at room temperature. Cells were permeabilized with 0.1% Triton X-100 (Sigma Aldrich) in phosphate-buffered saline (PBS) for 20 min and were then blocked with 1% bovine serum albumin (BSA) in PBS for 1 h. Slides were incubated with the primary antibodies anti-EGFR mouse monoclonal antibody (clone A-10, sc-373746; Santa Cruz Biotechnology, Santa Monica, CA, USA), anti-CYLD mouse monoclonal antibody (clone E-10, sc-137139; Santa Cruz Biotechnology), goat polyclonal anti-human-IgG (A80-119A; Bethyl Laboratories, Montgomery, TX, USA), or anti-hemagglutinin (HA)-tag antibody (clone F-7, sc-7342; Santa Cruz Biotechnology) for 1 h at room temperature, followed by incubation with Alexa Fluor 488-, 594- or 647-conjugated donkey anti-rabbit IgG (A-21206, A-21207), donkey anti-mouse IgG (A-21202, A-21203, A-31571), and donkey anti-goat IgG (A-11055) from Thermo Fisher Scientific at room temperature for 30 min in the dark. Nuclei were stained with 5 µg/mL Hoechst 33,342 (Sigma-Aldrich) in PBS. Slides were mounted and sealed with clear nail polish. Slides were stored at −20 °C in the dark until being observed with a microscope (FV3000; Olympus, Tokyo, Japan).

### 2.6. Measurement of Cell-Surface EGFR Expression

Cells were harvested and washed twice with PBS. Cells were fixed with 2% PFA at room temperature for 15 min and were then blocked for 10 min with an incubation buffer (PBS containing 0.1% BSA). Cells were resuspended in 100 μL of a buffer containing 0.5 μg of anti-EGFR antibody (clone LA1, Merck), a mouse IgG1 targeting the external domain of human EGFR, and were incubated for 1 h on ice. After cells were washed three times with the incubation buffer and resuspended in 100 μL of the incubation buffer with Alexa Fluor488-conjugated goat anti-mouse IgG (A-11029, Thermo Fisher Scientific) for 1 h on ice, cell-surface EGFR was analysed using FACSVerse (BD Biosciences, San Jose, CA, USA).

### 2.7. Apoptosis Assay

Cells were pretreated with nystatin (25 µg/mL), mβCD (10 mM), or CPZ (5 µM) for 30 min before treatment with 100 µg/mL of CTX. Cells were harvested and washed twice with PBS. Equal cell numbers were resuspended in tubes. Cells were stained with Annexin V-APC and 7-AAD (BD Biosciences). FACSVerse (BD Biosciences) was used to measure apoptosis.

### 2.8. Western Blotting

Cells were washed twice in cold PBS and were lysed by adding a NP-40 buffer containing Protease/Phosphatase Inhibitor Cocktail (Cell Signaling Technology, Tokyo, Japan). After a 30 min incubation of the lysate on ice, the lysate was centrifuged at 15,000 rpm for 15 min to remove insoluble substances. Protein concentrations were determined by using a BCA kit (Pierce Chemical Co., Rockford, IL, USA). Equal amounts of protein were fractionated via SDS-PAGE and transferred to PVDF membranes. The membranes were blocked with 5% skim milk and 0.1% Tween 20 (Sigma-Aldrich) in Tris-buffered saline (TBS) (pH 7.4), and were then incubated overnight at 4 °C with antibodies against CYLD (Santa Cruz Biotechnology), EGFR (Santa Cruz Biotechnology), pEGFR (Tyr1068, Cell Signaling Technology), pAKT (Ser473, Cell Signaling Technology), pERK (Thr202/Tyr204, Cell Signaling Technology), pSTAT3 (Tyr705, Cell Signaling Technology), AKT (Cell Signaling Technology), ERK (Cell Signaling Technology), STAT3 (Cell Signaling Technology), β-actin (Sigma-Aldrich), or HA (Santa Cruz Biotechnology) in 5% BSA/TBS. After being washed, the membranes were incubated in horseradish peroxidase (HRP)-conjugated secondary antibodies for 1 h. Protein bands were detected using an enhanced chemiluminescence system (Amersham Pharmacia Biotech, Buckinghamshire, UK). All the whole western blot figures can be found in the Supplementary Materials.

### 2.9. Samples from Patients and Patients’ Backgrounds

Tumour specimens were obtained from 29 patients with oral squamous epithelial cancer who underwent therapeutic surgery or biopsy at the Department of Oral and Maxillofacial Surgery, Kumamoto University Hospital, from 2002 to 2017. Fifteen patients had surgery before CTX administration. Eleven of these patients received TS-1 before and after surgery. Nine patients were inoperable and were given CTX as the initial treatment. Five patients received primary treatment that included radiation monotherapy and TS-1 before CTX. Seventeen of the 29 patients were switched from CTX to another drug because of tumour regrowth or reactions to the infusions. Various treatments were used after the switch, including 5-fluorouracil plus cisplatin, paclitaxel, and TS-1. Tissue samples for immunohistochemistry were fixed with 10% formalin before treatment. This study was approved by the Ethics Committee of Kumamoto University, and all subjects gave written informed consent to participate.

### 2.10. Immunohistochemistry

Paraffin-embedded formalin-fixed clinical tissues were cut to a thickness of 5 µm and were placed on glass slides. Tissues were dewaxed with xylene and then rehydrated in descending concentrations of alcohol. After the tissue antigen was activated with Proteinase K (DAKO, Jena, Germany), endogenous peroxidase was removed by incubation with 3% hydrogen peroxide for 15 min. To block non-specific background staining, tissues were incubated for 10 min with a non-specific staining blocking reagent (DAKO). Tissues were then incubated overnight at 4 °C with anti-CYLD mouse monoclonal antibody (clone E-10, sc-74435; Santa Cruz Biotechnology) or anti-EGFR mouse monoclonal antibody (clone A-10, sc-373746; Santa Cruz Biotechnology) diluted in PBS containing 1% BSA. Tissues were rinsed with PBS for 5 min and incubated for 1 h with HRP-conjugated secondary antibody. 3,3′-Diaminobenzidine (DAKO) was used for chromogen development. Tissues were counterstained with haematoxylin for 30 s, dehydrated, and mounted.

The CYLD staining score in clinical oral squamous cell carcinoma specimens was determined by using ImageJ software (National Institutes of Health, Bethesda, MD, USA). Regions of interest were drawn manually at 200× magnification, and then the percent positive area in each region of interest was determined and scored by using the threshold tool of the software, with a CYLD-negative stromal lesion used as the negative control: i.e., score 0, <5%; score 1, 5–25%; score 2, 25–50%; score 3, 50–75%; score 4, >75%. No case in this study had a CYLD staining score of 4. The samples were classified into two categories according to the CYLD expression: low (scores of 0–1) or high (scores of 2–3). The EGFR staining pattern was evaluated according to its subcellular distribution in cancer cells; the pattern of the EGFR signal that was observed primarily on the cell membrane was defined as “membrane EGFR” for individual cancer cells. Other staining patterns included EGFR signals in the cytoplasm or both the cytoplasm and the cell membrane. The average percentage of cells showing the membrane EGFR pattern viewed in several fields at 200× magnification was calculated, and a membrane EGFR staining score (i.e., scores of 0–4) was assigned using the same classification criteria as the CYLD staining score described above. Scores of 0–1 and 2–4 were defined as low and high, respectively.

### 2.11. Statistical Analysis

Statistical significance was defined as *p* < 0.05 using Student’s paired t-test (to compare the means of two groups) and Pearson’s χ^2^ test. JMP software Version 13 for Windows (SAS Institute, Cary, NC, USA) was used for statistical analysis.

### 2.12. Flow Diagram

There is a flow diagram of this study in Figure S1.

## 3. Results

### 3.1. CME and Degradation of EGFR Are Essential for CTX-Induced Apoptosis

**Figure 1:**
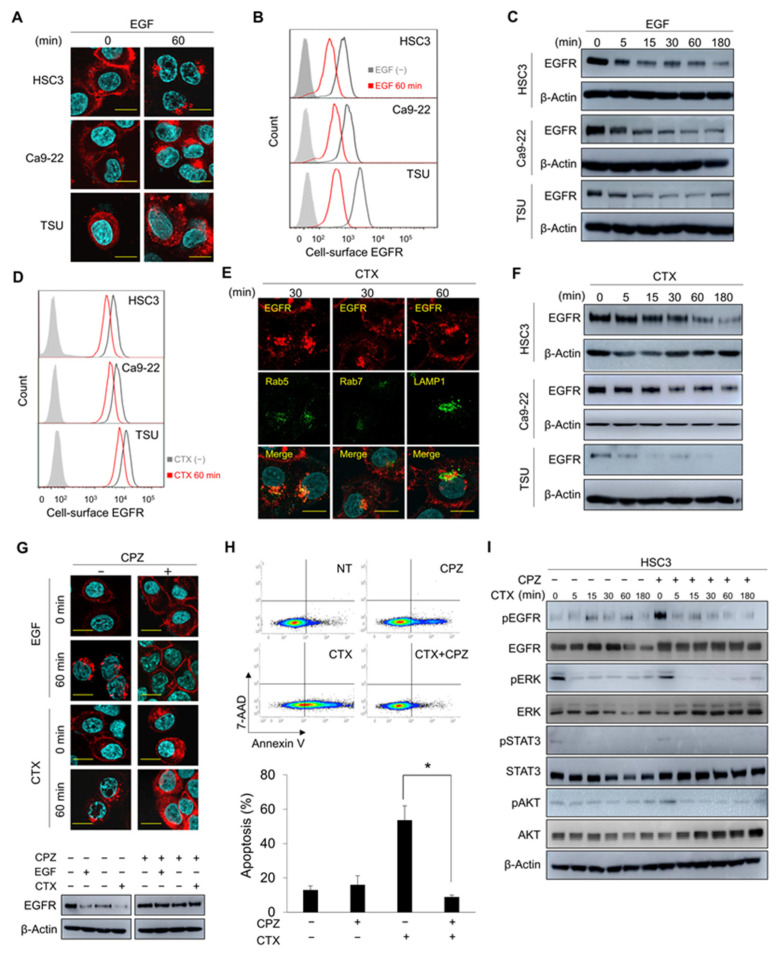
CME of EGFR is essential for CTX-induced apoptosis. (**A**,**B**) Localization of EGFR after EGF stimulation. HSC3, Ca9-22, and TSU cells were stimulated with 100 ng/mL EGF for 60 min. (**A**) EGFR localization was analysed via immunofluorescence staining. Scale bars, 10 μm. (**B**) Cell-surface EGFR was analysed using flow cytometry. (**C**) Amount of total EGFR after EGF stimulation. Cells were stimulated with 100 ng/mL EGF for the indicated times and total EGFR expression was analysed by using Western blotting. (**D**) Cell-surface EGFR expression after CTX treatment. Cells were treated with 100 μg/mL CTX for 60 min, and then cell-surface EGFR was stained with an anti-EGFR antibody (clone LA1). (**E**) Co-localization of EGFR with endosomes and lysosomes after CTX treatment. The localizations of EGFR and the endosome and lysosome markers were analysed via immunofluorescence staining after 100 μg/mL CTX treatment for 30 min (Rab5 and Rab7) or 60 min (LAMP1). Scale bars, 10 μm. (**F**) Amount of total EGFR expression after EGF stimulation. Cells were stimulated with 100 μg/mL CTX for the indicated times and total EGFR expression was analysed by using Western blotting. (**G**) Effects of CPZ on EGFR internalization and degradation after EGF or CTX treatment. HSC3 cells were pretreated with 5 μM CPZ for 30 min and were then stimulated with 100 ng/mL of EGF or 100 μg/mL of CTX for 60 min. CHX was added before adding CPZ. The localization of EGFR was analysed via immunofluorescence staining (upper panels). Total EGFR expression was analysed via Western blotting (lower panels). Scale bars, 10 μm. (**H**) Apoptosis after CTX given with CPZ. HSC3 cells were cultured in the presence of 5 μM CPZ for 30 min, followed by incubation with 100 μg/mL of CTX for 12 h in serum-free medium. Cells were harvested and stained with Annexin V-APC and 7-AAD. NT, no treatment. Bars indicate the percentage of apoptotic cells. * *p* < 0.05. (**I**) Phosphorylation of EGFR and major downstream molecules after CTX given with CPZ. Cells were pretreated with 5 μM CPZ for 30 min, after which they were treated with 100 μg/mL of CTX for the indicated times.

We first evaluated the response of EGFR to EGF stimulation in the human HNSCC cell lines HSC3, Ca9-22, and TSU. EGF stimulation induced EGFR endocytosis (Figure 1A) and reduced cell-surface EGFR expression (Figure 1B). Most of the endocytosed EGFR co-localized with the early endosome marker Rab5 at 30 min, and with the late endosome marker Rab7 [37] and the lysosome marker LAMP1 at 60 min after EGF addition (Figure S2A). After EGF stimulation, EGFR protein levels decreased markedly (Figure 1C and Figure S2A), which confirmed the degradation of EGFR protein in lysosomes [38]. We then investigated the effects of CTX on EGFR expression in these cell lines. Similar to EGF stimulation, CTX treatment led to reduced cell-surface EGFR expression (Figure 1D), and a subsequent intracellular co-localization of EGFR with early and late endosomes and lysosomes (Figure 1E). Thus, EGFR degradation occurred (Figure 1F).

Binding of the ligands and CTX to EGFR induced conformational changes in EGFR that formed dimers and triggered EGFR endocytosis, which was mediated by clathrin [24,38]. Consistent with these reports, the endocytosis of EGFR induced by EGF and CTX was blocked by the clathrin inhibitor CPZ (Figure 1G, upper panels, Figure S2B). The CHX chase assay thus showed that pretreatment with CPZ inhibited the EGF- and CTX-induced degradation of EGFR (Figure 1G, lower panels). As an important result, CTX-induced apoptosis was completely blocked by CPZ pretreatment (*p* = 0.011, Figure 1H), although phosphorylation of EGFR and the major downstream effectors, including STAT3, AKT, and ERK, was inhibited (Figure 1I). Together, these data indicate that CTX-induced CME and degradation of EGFR were crucial for apoptosis induction in HNSCC cells rather than CTX inhibition of the EGFR signalling pathway.

### 3.2. CYLD Is Required for EGF- and CTX-Induced CME of EGFR

**Figure 2:**
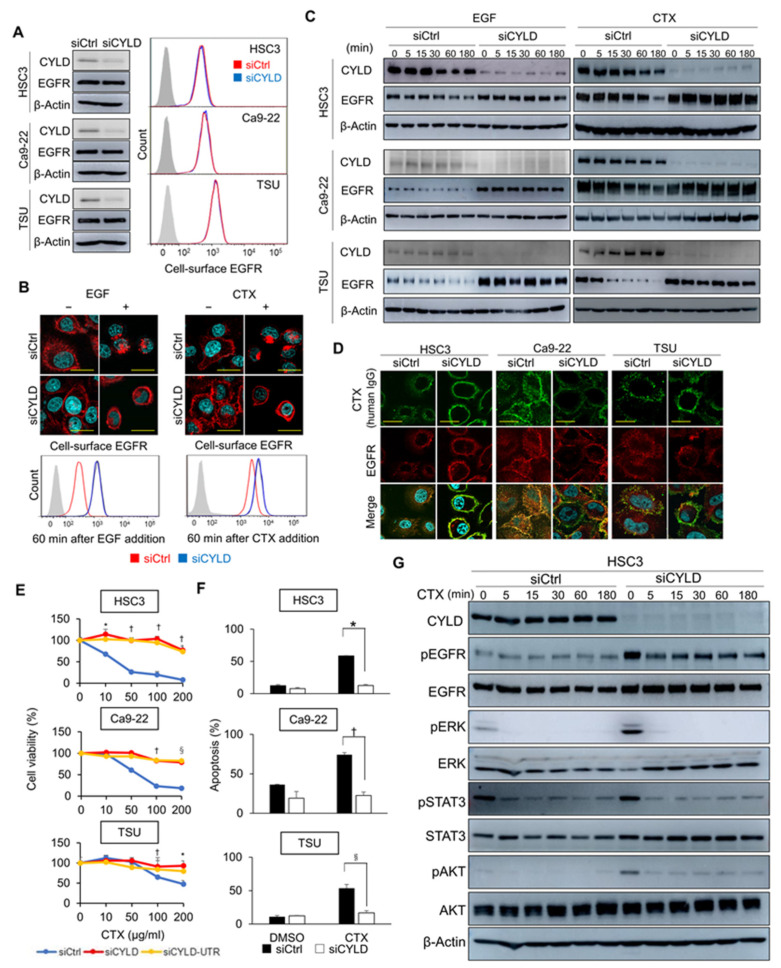
CYLD downregulation inhibits EGF- and CTX-induced CME of EGFR. (**A**) CYLD and EGFR expression after CYLD knockdown. HSC3, Ca9-22, and TSU cells were transfected with siRNA and then incubated for 48 h. The expression of total CYLD and EGFR was analysed by using Western blotting (left panels). Cell-surface EGFR expression was analysed by means of flow cytometry (right panels). (**B**) Internalization of EGFR after EGF or CTX treatment in CYLD-downregulated cells. HSC3 cells were treated with 100 ng/mL of EGF or 100 μg/mL of CTX for 60 min. EGFR localization was analysed via immunofluorescence staining (upper panels). Cell-surface EGFR expression was analysed via flow cytometry (lower panels). Scale bars, 10 μm. (**C**) Total EGFR expression levels after treatment with EGF or CTX in CYLD-downregulated cells. HSC3, Ca9-22, and TSU cells were transfected with siRNA and were then stimulated with 100 ng/mL of EGF or 100 μg/mL of CTX for the indicated times before harvesting. The cell lysate was immunoblotted with antibodies against the indicated proteins. (**D**) EGFR and CTX localization in CYLD-downregulated cells. HSC3, Ca9-22, and TSU cells were transfected with siRNA and were then treated with 100 μg/mL of CTX for 60 min. Immunofluorescence staining was used to analyse the localization of EGFR and CTX. Scale bars, 20 μm. (**E**) Cell viability after CTX treatment in CYLD-downregulated cells. HSC3, Ca9-22, and TSU cells were transfected with siRNA for 48 h before treatment with 100 μg/mL of CTX for 72 h in serum-free medium. * *p* < 0.05; † *p* < 0.01; ^§^ *p* < 0.005 (siCtrl vs. siCYLD or siCYLD-UTR). (**F**) Apoptosis after CTX treatment in CYLD-downregulated cells. HSC3, Ca9-22, and TSU cells were transfected with siRNA and incubated for 48 h. The medium was changed to serum-free medium and then 100 μg/mL of CTX was added. After a 12 h incubation with CTX, cells were harvested and analysed by means of Annexin V-APC and 7-AAD. * *p* < 0.01; † *p* < 0.001; ^§^ *p* < 0.005. (**G**) Phosphorylation changes in EGFR and the downstream signalling molecules after treatment with CTX in CYLD-downregulated cells. HSC3 cells were transfected with siRNA and incubated for 48 h. The medium was changed to serum-free medium, and cells were then treated with 100 μg/mL of CTX for the indicated times. Cell lysates were immunoblotted with antibodies against the indicated proteins.

The molecular mechanisms involved in EGFR signalling have been analysed extensively. Nevertheless, the critical factors regulating the CME of EGFR, especially those commonly induced by the ligand and CTX, are largely unknown. We previously reported that downregulation of CYLD protein expression frequently occurs in primary HNSCC tissues [34]. However, the roles of CYLD in EGFR signalling remain unclear. Therefore, we studied the effects of downregulating CYLD expression on EGFR trafficking through transfection with siRNA to target the CYLD gene coding region (siCYLD). The basal expression levels of total and cell-surface EGFR protein showed no apparent differences in cells transfected with control siRNA (siCtrl) or siCYLD (Figure 2A). However, CYLD knockdown strongly inhibited the EGF- and CTX-induced CME of EGFR (Figure 2B, upper panels), and thus cell-surface EGFR expression remained unaltered in CYLD-downregulated cells (Figure 2B, lower panels). Moreover, CYLD knockdown inhibited EGF- and CTX-induced EGFR degradation (Figure 2C). In addition, flow cytometric analysis with anti-human IgG demonstrated the suppression of CTX internalization in CYLD-downregulated cells (Figure S3A). Our immunofluorescence staining consistently showed that CTX remained at the cell membranes with EGFR in CYLD-downregulated cells, whereas CTX co-localized mostly with endocytosed EGFR in the cytoplasm in cells transfected with siCtrl (Figure 2D).

Given our observation that CTX’s efficacy was attributed to efficient endocytosis and the degradation of EGFR (Figure 1), we analysed the effects of CYLD downregulation on CTX sensitivity. CYLD knockdown with different two siRNAs led to resistance to CTX (Figure 2E and Figure S3B) because of complete inhibition of apoptosis induction (HSC3: *p* = 0.006; Ca9-22: *p* = 0.0007; TSU: *p* = 0.0032) (Figure 2F). Loss of CYLD increased the basal phosphorylation levels of EGFR and the major downstream effectors, including STAT3, AKT, and ERK (Figure 2G). However, CTX effectively blocked their phosphorylation even in CYLD-downregulated cells, as also observed in siCtrl-transfected cells (Figure 2G). These data suggested that suppression of the activity of these major effectors by CTX was independent of endocytic EGFR trafficking, at least in the short term, and that EGFR endocytosis was more important for the antitumour effects of CTX than the inhibition of EGFR signalling. Our data thus indicate that CYLD is essential for the CME and degradation of EGFR induced by EGF and CTX, and for the induction of apoptosis by CTX in HNSCC cells.

### 3.3. The N-Terminal Part of CYLD Is Responsible for EGFR CME and CTX-Induced Apoptosis

**Figure 3:**
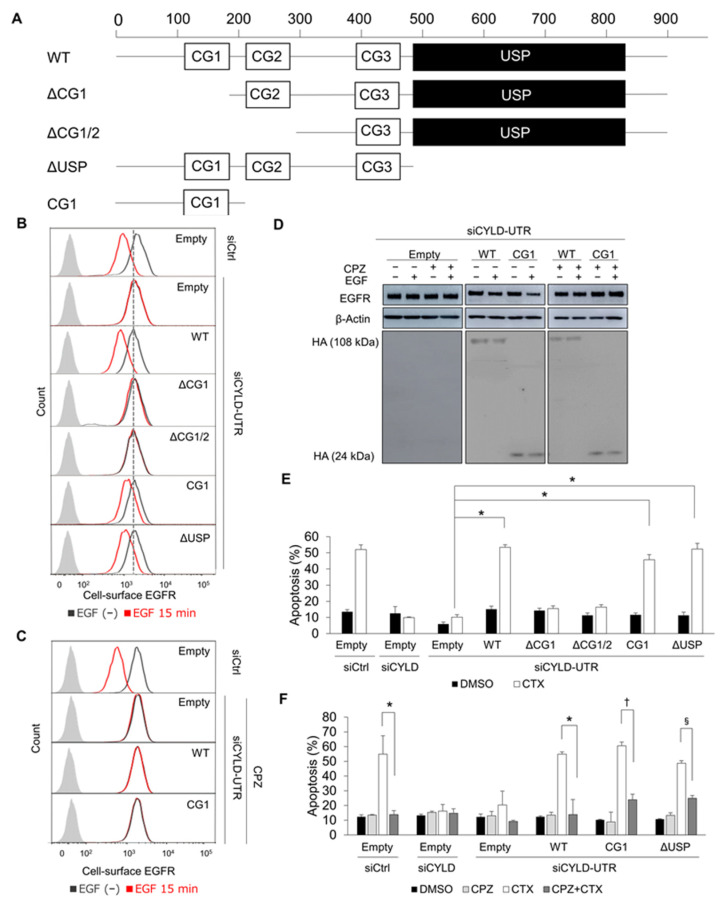
Impact of CYLD domain deficiency on CTX’s efficacy. (**A**) Domain structure of human CYLD protein and constructs encoding deletion mutants of CYLD. Three CG domains (CG1–3) and the USP domain are shown as white and black boxes, respectively. (**B**) The effects of CYLD mutants on cell-surface EGFR expression after EGF stimulation. HSC3 cells were co-transfected with CYLD deletion mutants and siRNA. After a 48 h incubation, cells were stimulated with 100 ng/mL of EGF for 15 min. Flow cytometry was used to analyse cell-surface EGFR expression. (**C**) The effects of WT-CYLD and CG1 constructs on cell-surface EGFR expression after EGF stimulation in combination with CPZ. HSC3 cells were co-transfected with WT-CYLD or CYLD mutants and siRNA. After a 48 h incubation, cells were stimulated with 100 ng/mL of EGF for 15 min. CPZ (5 μM) was added 30 min before adding the EGF. Flow cytometry was used to analyse cell-surface EGFR expression. (**D**) The effects of CYLD mutants on total EGFR expression. HSC3 cells were co-transfected with WT-CYLD or CG1 constructs and siRNA. After incubation for 48 h, the medium was changed to a serum-free medium and then the incubation continued for 12 h. Cells were stimulated with 100 ng/mL of EGF or 100 μg/mL of CTX for 60 min and were analysed by immunoblotting. (**E**,**F**) The effects of CYLD mutants on apoptosis induced by CTX. HSC3 cells were co-transfected with WT-CYLD or CYLD deletion constructs and siRNA. After incubation for 12 h with 100 μg/mL of CTX in serum-free medium, cells were harvested and analysed with Annexin V-APC and 7-AAD. * *p* < 0.001 (**E**). CPZ (5 μM) was added 30 min before adding CTX. * *p* < 0.05; † *p* < 0.01; ^§^ *p* < 0.0001 (**F**).

We next analysed EGFR trafficking in HSC3 cells concomitantly transfected with various CYLD deletion constructs (Figure 3A) and siCYLD-UTR. Cell-surface EGFR expression did not differ significantly among cells transfected with the deletion constructs (Figure 3B). As expected, transfection of full-length wild-type (WT)-CYLD in siCYLD-UTR-transfected cells restored EGFR internalization after EGF or CTX stimulation (Figure 3B and Figure S4A). Transfection of a construct lacking the USP domain (ΔUSP) also restored EGFR internalization. However, both ΔCG1 and ΔCG1/2 failed to rectify impaired EGFR trafficking. Thus, transfection of the N-terminal part containing the CG1 domain alone was enough to restore EGFR internalization. Again, clathrin mediated this recovery (Figure 3C and Figure S4B). Transfection with CG1 also restored EGFR degradation after EGF stimulation in a clathrin-dependent manner (Figure 3D). Although the amount of early reduction in cell-surface EGFR was smaller in CG1- and ΔUSP-transfected cells than in WT-transfected cells (Figure 3B and Figure S4A), we found effective EGFR degradation (Figure 3D), which suggested the involvement of the USP domain in the facilitation of EGFR internalization. Since CME of EGFR was essential for CTX-induced apoptosis (Figure 1), we next investigated apoptosis. No apparent difference in basal apoptosis was found among cells transfected with the deletion constructs (Figure 3E). Consistent with the data for EGFR trafficking, HSC3 cells co-transfected with siCYLD-UTR and full-length WT-CYLD, ΔUSP, or CG1 showed effective induction of apoptosis after CTX (WT-CYLD: *p* = 0.00019, ΔUSP: *p* = 0.00048, CG1: *p* = 0.00049). Transfection of the CYLD p.C601A mutant lacking deubiquitinase activity also restored CTX’s efficacy (Figure S4C). Clathrin activity was essential for the recovery of CTX-induced apoptosis by CG1 (Figure 3F). All our data showed that the CG1-containing the N-terminal part rather than deubiquitinase activity was responsible for CYLD regulation of CME and the degradation of EGFR induced by EGF and CTX, and thus efficient induction of apoptosis by CTX.

### 3.4. Relationship between CYLD Expression and Subcellular EGFR Localization in Human HNSCC Tissues

**Figure 4:**
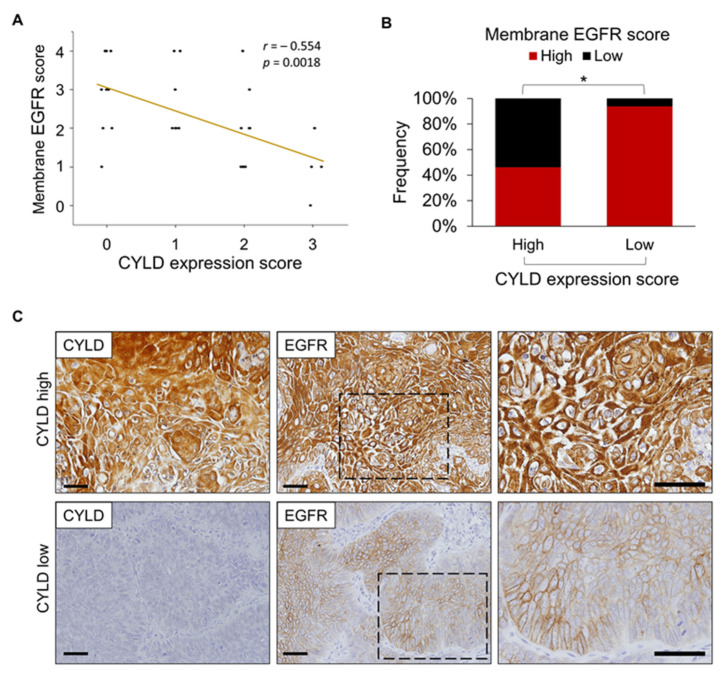
CYLD expression and subcellular EGFR localization in human HNSCC tissues. (**A**) Relationship between CYLD expression score and membrane EGFR score in primary HNSCC tissues. (**B**) Percentage of specimens with low or high CYLD expression scores compared with membrane EGFR scores. The membrane EGFR score was determined based on the percentage of tumour cells showing dominant EGFR localization in the cell membranes (see Materials and Methods for scoring details). * *p* < 0.01 (Pearson’s χ^2^ test). (**C**) Examples of high CYLD expression for low membrane EGFR scores and low CYLD expression for high membrane EGFR scores. The images on the right provide enlargements of the boxed areas in the middle images. Scale bars, 100 μm.

We used immunohistochemistry with anti-CYLD and anti-EGFR antibodies to study the initial biopsy samples from 29 primary HNSCC patients who underwent CTX-based therapies. Similar to the results in our previous reports [34], tumour cells in more than half of the cases (16/29) showed lower CYLD protein expression levels (score 0–1, see Materials and Methods), with this expression being absent (score = 0) in 31% (9/29) of cases (Figure S5A). Tumour cells in all cases expressed EGFR. We observed intratumour heterogeneity in subcellular EGFR localization. We therefore placed the samples into five groups (scores of 0–4) according to the percentage of tumour cells in which EGFR was localized primarily to the cell membranes (see Materials and Methods). CYLD expression was inversely correlated with membrane EGFR expression (Figure 4A). Almost all cases with low CYLD scores showed high membrane EGFR scores (15/16 cases, 94%); 46% (6/13 cases) with high CYLD scores showed high membrane EGFR scores (Figure 4B,C). CYLD localized to both the cytoplasm and cell membrane in tumour cells (Figure 4C). Intracellular EGFR accumulation frequently occurred in CYLD-expressing tumours, which suggested active endocytosis. Although we found no statistical correlation between CYLD expression or membrane EGFR score and therapeutic efficacy as assessed by the progression-free survival rate or the overall survival rate (Figure S5B), these results were likely due to inconsistent therapeutic regimens across patients (see Materials and Methods) and the limited cohort size. Our immunohistochemical observations support in vitro data indicating that downregulation of CYLD inhibited the intracellular trafficking of EGFR after ligand stimulation.

### 3.5. Cholesterol Sequestration Restores CYLD Knockdown-Induced Defective EGFR Trafficking and Overcomes CTX Resistance

**Figure 5:**
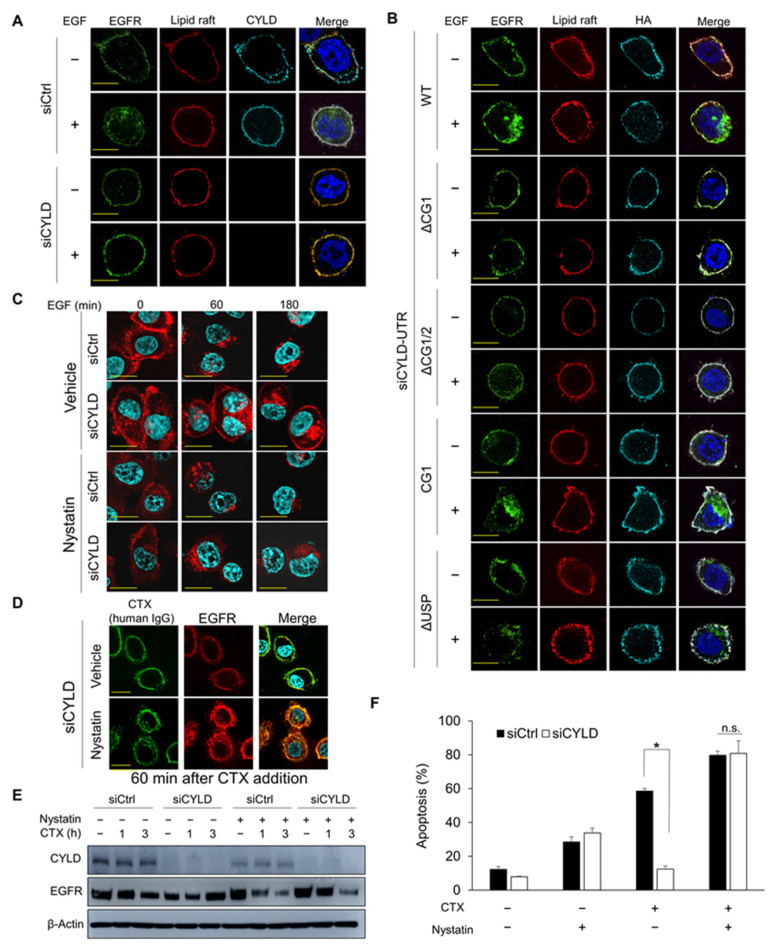
Effect of cholesterol depletion on CYLD-downregulated cells. (**A**,**B**) Localization of EGFR, lipid rafts, and CYLD (**A**) or anti-hemagglutinin (HA) (**B**) as analysed by immunofluorescence staining. HSC3 cells were transfected with the indicated siRNA and plasmids expressing deletion constructs of CYLD. After incubation for 48 h, cells were starved for 12 h in a serum-free medium. Cells were then stained with the appropriate antibodies and observed under fluorescent microscopy. Scale bars, 10 µm. (**C**,**D**) Effects of nystatin on EGF- or cetuximab (CTX)-induced EGFR endocytosis. HSC3 cells were transfected with siRNA and incubated for 48 h, then, after 12 h of incubation in a serum-free medium, cells were pretreated with 25 µg/mL of nystatin for 30 min before stimulation with 100 ng/mL of EGF for the indicated times (**C**) or 100 µg/mL of CTX for 60 min (**D**). EGFR localization was analysed via immunofluorescence staining. Scale bars, 20 µm. (**E**) Effects of nystatin on total EGFR expression after CTX treatment in CYLD-downregulated cells. HSC3 cells were transfected with siRNA and were then incubated for 48 h. Cells were pretreated with 25 µg/mL of nystatin for 30 min before treatment with 100 µg/mL of CTX for the indicated times. Total EGFR protein expression was analysed via Western blotting. CHX was added 1 h before nystatin treatment. (**F**) Effects of nystatin on CTX-induced apoptosis. HSC3 cells were transfected with siRNA and then incubated for 48 h. Cells were pretreated with 25 µg/mL of nystatin for 30 min before treatment with 100 µg/mL of CTX in a serum-free medium for 12 h. Apoptosis was analysed using Annexin V-APC and 7-AAD. * *p* < 0.001; n.s., not significant.

Lipid rafts are microdomains of plasma membranes that are greatly enriched with cholesterol and sphingolipids and regulate the function of many surface receptors [39]. EGFR initially located in lipid rafts may exit these rafts after ligand binding and enter clathrin-coated pits [40]. CYLD reportedly co-localized with lipid rafts in T cells [41]. To clarify how CYLD regulates EGFR internalization, we investigated the subcellular localization of endogenous CYLD and hemagglutinin (HA)-tagged CYLD deletion constructs in relation to EGFR and lipid rafts. In cells transfected with siCtrl, both the cytoplasm and plasma membranes possessed endogenous CYLD (Figure 5A). Cell-surface CYLD and EGFR mostly co-localized with lipid rafts in cells without EGF stimulation. As expected, after EGF stimulation, EGFR exited the lipid rafts and was internalized, without an apparent alteration in CYLD distribution. However, in cells transfected with siCYLD, EGFR remained with the lipid rafts in plasma membranes even after EGF stimulation. Consistent with the data in Figure 3B, EGFR internalization was restored only when the CG1-containing N-terminal part of CYLD was expressed (Figure 5B). Our immunofluorescence analysis showed that all HA-tagged CYLD deletion constructs, including those lacking the N-terminal part, co-localized with lipid rafts without affecting basal EGFR localization. These findings indicate that the CG1-containing N-terminal part of CYLD in lipid rafts was essential for EGFR to exit the lipid rafts and that inhibition of CME of EGFR by the loss of CYLD was attributed to restricting EGFR to the lipid rafts.

Disruption of the lipid rafts by cholesterol depletion induced the trafficking of EGF- or CTX-bound EGFR to clathrin-coated pits, thus enhancing CME [42,43]. The involvement of cholesterol accumulation in cancer aggravation was also suggested in a colon cancer model [44]. However, the effects of cholesterol reduction on impaired CME remain unknown. To determine whether cholesterol reduction enables the release of EGFR confined to lipid rafts and affects sensitivity to CTX, we treated cells with nystatin or mβCD, which reduce cholesterol in lipid rafts/caveolae [45]. Pretreatment with nystatin (Figure 5C and Figure S6A) or mβCD (Figure S6A,B) restored EGF-induced CME of EGFR in CYLD-downregulated cells. Moreover, nystatin or mβCD restored the internalization of CTX-bound EGFR in CYLD-downregulated cells (Figure 5D and Figure S6C). In agreement with these data, nystatin restored CTX-induced EGFR degradation in CYLD-downregulated cells (Figure 5E). Nystatin also promoted the degradation of CTX-bound EGFR in siCtrl-transfected cells (Figure 5C,E). Cholesterol depletion with both agents fully restored sensitivity to CTX in CYLD-downregulated cells; nystatin increased basal sensitivity to CTX (*p* = 0.0006, Figure 5F). mβCD also restored sensitivity to CTX in CYLD-downregulated cells (Figure S6D). These data indicate that cholesterol reduction enabled the release of EGFR from lipid rafts to clathrin-coated pits, thereby overcoming resistance to CTX in CYLD-downregulated cells.

## 4. Discussion

The molecular mechanisms of trafficking of ligand-bound EGFR have been well documented. However, those for CTX-induced EGFR trafficking remain largely unknown despite EGFR’s modulation of antitumour activity. We show here that the tumour suppressor CYLD is essential for CME of EGFR and its lysosomal degradation is induced by EGF and CTX in HNSCC cells. Regulation of EGFR trafficking by CYLD is necessary for CTX’s apoptosis-inducing effect.

We found that the N-terminal part of CYLD containing the CG1 domain was responsible for EGF- and CTX-induced CME and the subsequent degradation of EGFR in HNSCC cells. Our data showed that CYLD loss inhibited EGFR’s exit from lipid rafts to clathrin-coated pits. A substantial fraction of CYLD was in the lipid rafts. CYLD reportedly co-localized with lipid rafts in CD3-positive T cells under resting conditions but not in their protein kinase Cθ (PKCθ)/β-deficient counterparts [41], which suggests a PKC-dependent membrane shuttling of CYLD. Although the detailed mechanisms underlying the localization of CYLD in lipid rafts remain to be clarified, we discovered that all CYLD mutants tested, including those without the N-terminal part, localized in lipid rafts, which suggests that regulation of EGFR internalization did not depend only on CYLD’s location but also on the N-terminal part of CYLD, which has some effects on lipid rafts. Although phosphorylation of CYLD by EGF at the Tyr-15 upstream of CG1 reportedly promoted the recruitment of Cbl-b to activated EGFR and EGF-induced EGFR trafficking [46], this mechanism would not explain the behaviour of inactive CTX-bound EGFR that we observed. Among the three CG domains of CYLD protein, CG1 and CG2 interacted directly with tubulin and microtubules, and CG1 had the highest binding affinity [42,47]. CG1 also interacted with histone deacetylase 6, which deacetylates various proteins, including tubulin [48]. These data suggest the involvement of tubulin dynamics in the release of EGFR from lipid rafts to clathrin-coated pits. Tubulin resides in lipid rafts and clathrin-coated pits, and its modifications, including acetylation, alter the localization of some proteins [49,50]. Thus, investigating the interacting partners of the N-terminal part of CYLD, especially CG1, and modification of the tubulin in lipid rafts would be important.

In terms of the treatment of cancers such as HNSCC, distinct from other cancers including CRC and NSCLC, the factors contributing to primary resistance to CTX remain poorly understood [14]. Here, we demonstrated that CYLD loss induced CTX resistance via inhibition of CME of EGFR and that the N-terminus of CYLD was involved in this phenomenon. Reduced CYLD expression is frequently found, predominantly in invasive lesions in primary HNSCC [51], which may at least partly explain why CTX is not as effective in this cancer as one would expect from preclinical data [11]. Our immunohistochemical findings suggest that membrane expression of EGFR may partly reflect the impaired trafficking of this receptor. Although acquired resistance to CTX often involves persistent activation of the signalling effectors downstream of EGFR [51], downregulation of CYLD did not block the inhibitory effects of CTX against major downstream effectors, which suggests that EGFR internalization/degradation, rather than the inhibition of downstream signalling, is critical for CTX-induced apoptosis. Indeed, the downregulation of cell-surface EGFR after CTX treatment reportedly predicted antitumour effects in CRC [26]. Moreover, impaired endocytosis of CTX-bound EGFR is a common biological feature in CTX-resistant NSCLC and HNSCC cells [52,53]. Therefore, reversing defective EGFR trafficking machinery may be crucial for improving the apoptotic activity of CTX.

We showed, as a promising example, that lipid raft disruption by cholesterol sequestration restored the CME of EGFR, thereby overcoming the CTX resistance induced by CYLD downregulation. This result suggests that cholesterol depletion effectively releases the EGFR in lipid rafts to clathrin-coated pits. Previous studies showed that nystatin promoted CTX-induced CME of EGFR and hence potentiated antitumour efficacy [36], as we observed. This drug is a polyene antifungal agent used both orally and topically in humans [54]. Another cholesterol-lowering drug, simvastatin, suppressed HNSCC growth ex vivo, enhanced the cytostatic effects of chemotherapeutics (cisplatin and docetaxel) [55], and even overcame CTX resistance in KRAS-mutant CRC by modulating BRAF activity [56]. In addition, high membrane cholesterol content itself inhibited EGFR internalization by accumulating specific factors in cholesterol-induced lipid rafts [57]. Although the regulation of EGFR trafficking by CYLD was independent of its deubiquitinase activity, a CYLD downregulation-induced reduction in this activity led to activation of diverse signalling pathways, including NF-κB and Wnt, thereby promoting malignant phenotypes [34]. Given that targeting such aggressive tumour cells is particularly important for establishing effective therapeutic strategies, CTX-based therapy combined with cholesterol-lowering drugs as a drug repositioning strategy may hold promise for HNSCC treatment.

## 5. Conclusions

In conclusion, we demonstrated that CYLD regulated the CME and degradation of EGFR induced by EGF and CTX via its N-terminal part. This process was required for CTX-induced apoptosis. Our data suggest that releasing EGFR from lipid rafts by sequestering cholesterol is useful for restoring CME of EGFR, thereby overcoming CTX resistance. Of note, in addition to direct EGFR inhibition, the antitumour activity of CTX depends on antibody-dependent cell cytotoxicity effects [58,59]. Because CYLD is implicated in the regulation of immunity [60], alterations of the immune microenvironment and their impacts on CTX efficacy are worth investigating. Our findings provide novel insights into the molecular mechanisms underlying EGFR trafficking and resistance to CTX. Additional studies will contribute to developing a novel treatment strategy targeting EGFR for HNSCC.

## Figures and Tables

**Table 1:** Primers used to construct CYLD deletion mutants.

Mutants	Forward Primers (5′–3′)	Reverse Primers (5′–3′)
ΔCG1	ATGCAGGTCGAACTTCCTCCTTTGG	GGGCCGGCCAGCGTAGTCTGGTACA
ΔCG1/2	ATGCTTGCCTTTATGTCAAGAGGTG	GGGCCGGCCAGCGTAGTCTGGTACA
CG1	GGCGCGCCTCTAGAACTATAGTGAG	TTATGCAGTGTCATCATCTTCTAT
ΔUSP	GGCGCGCCTCTAGAACTATAGTGAG	TTAAATCATTATCTCCAAGCCTTC

## Data Availability

The data presented in this study are available on request from the corresponding author.

## Supplementary Materials

The following supporting information can be downloaded at: https://www.mdpi.com/article/10.3390/cancers14010173/s1. Figure S1: Flow diagram of the method. Figure S2: EGFR trafficking after EGF or CTX treatment. Figure S3: Effects of CYLD knockdown on cell-surface CTX levels. Figure S4: Effects of various CYLD deletion mutants on cell-surface EGFR expression and cell viability. Figure S5: Expression of CYLD and EGFR in primary HNSCC tissues. Figure S6: Effects of mβCD on EGFR trafficking and CTX-induced apoptosis in CYLD-downregulated cells. Figures S7 and S8: Whole blots for the Western blots in Figure 1. Figures S9 and S10: Whole blots for the Western blots in Figure 2 and Figure S3. Figure S11: Whole blots for the Western blots in Figure 3 and Figure 5.
