# Supplementary material for: The Tumour Suppressor CYLD Is Required for Clathrin-Mediated Endocytosis of EGFR and Cetuximab-Induced Apoptosis in Head and Neck Squamous Cell Carcinoma
Source: Cancers (Basel). 2021 Dec 30;14(1):173. doi: 10.3390/cancers14010173 (PMC8750287; doi:10.3390/cancers14010173)
Supplement: Supplementary file 1 [file cancers-14-00173-s001.zip › cancers-1508300-supplementary.pdf]

Supplementary Figures

Figure S1

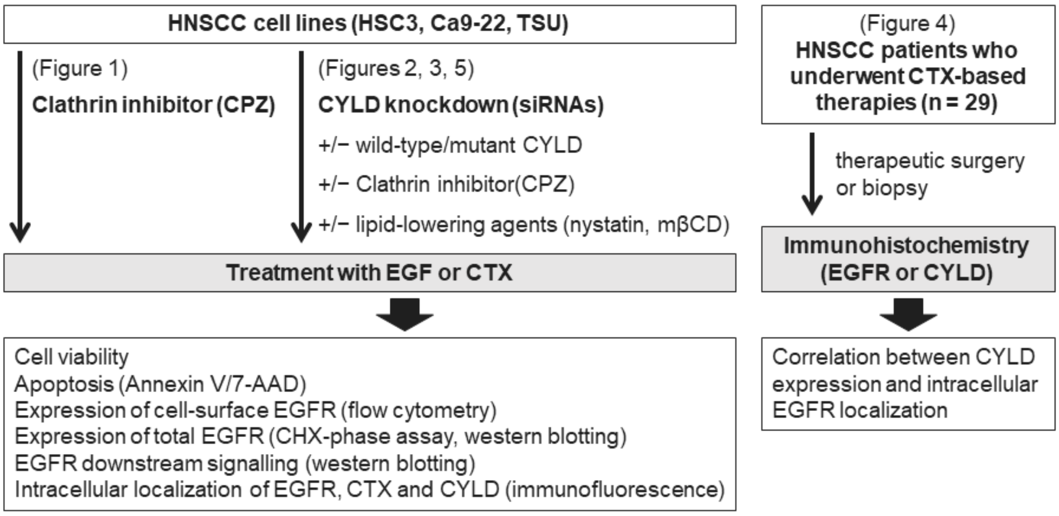

Figure S1. Flow diagram of the method.

The figure shows a diagram of the series of assays performed in this study.

Figure S2

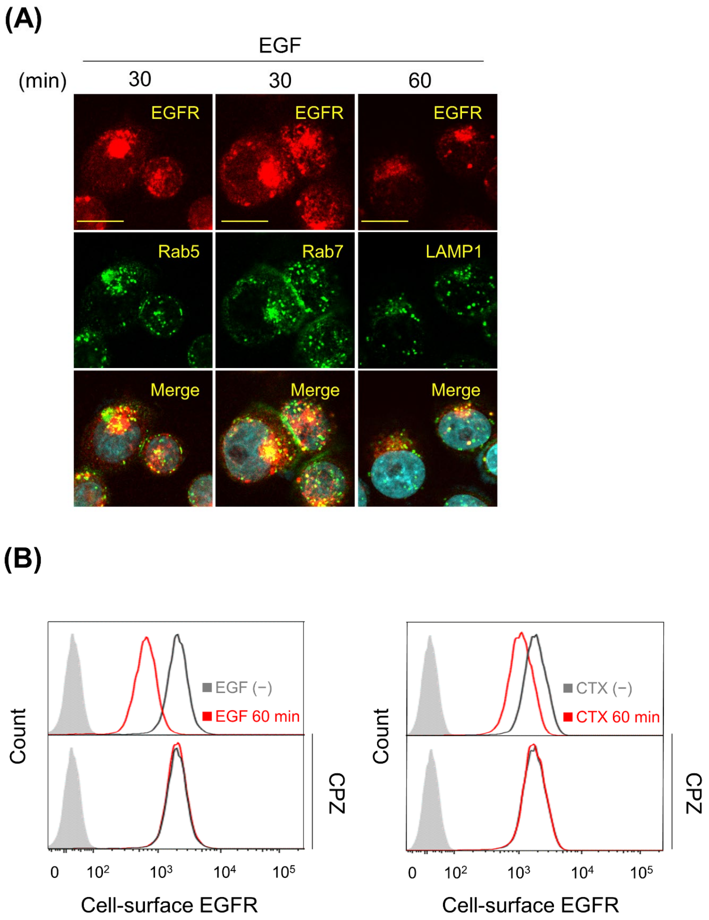

Figure S2. EGFR trafficking after EGF or CTX treatment.

(A) Localization of EGFR, endosome markers Rab5 and Rab7, and lysosome marker LAMP1 after EGF stimulation as analyzed by immunofluorescence staining. HSC3 cells were stimulated with 100 ng/ml EGF for 30 min (Rab5 and Rab7) or 60 min (LAMP1). Scale bars, 10 μm. (B) CPZ inhibited EGF- and CTX-induced EGFR endocytosis. HSC3 cells were stimulated with 100 ng/ml EGF or 100 μg/ml CTX for 60 min, and then cell-surface EGFR was analyzed by using flow cytometry. CPZ (5 μM) was added 30 min before stimulation with EGF or CTX.

Figure S3

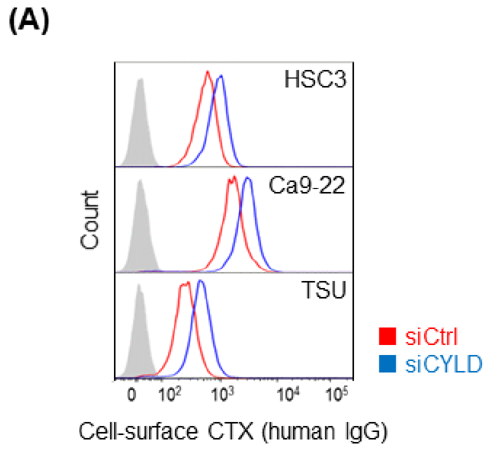

Figure S3. Effects of CYLD knockdown on cell-surface CTX level.

(A) HSC3 cells were transfected with siRNA. After a 48-h incubation, cells were starved for 12 h before addition of 100 μg/ml CTX. Cell-surface CTX was analyzed by flow cytometry. (B) Confirmation of CYLD knockdown by transfection with siCYLD-UTR. HSC3 cells were transfected with siCYLD-UTR. After a 48-h incubation, the cells were harvested and analyzed by Western Blotting.

Figure S4

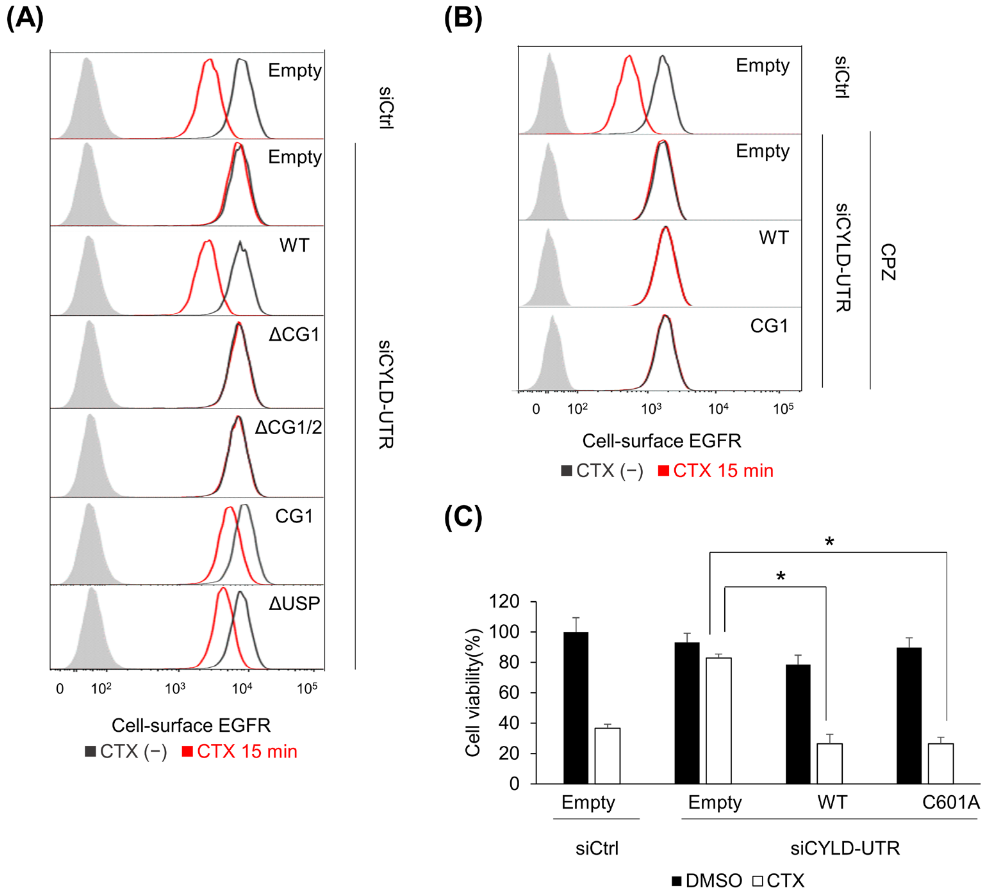

Figure S4. Effects of various CYLD deletion mutants on cell-surface EGFR expression and cell viability.

(A) Effects of CYLD mutants on cell-surface EGFR expression. HSC3 cells were co-transfected with siCYLD-UTR and CYLD deletion mutants. After incubation for 48 h, cells were starved in serum-free medium for 12 h. After 100 μg/ml CTX treatment for 15 min, cells were harvested, and cell-surface EGFR was analyzed by using flow cytometry. (B) Dependence of CTX-induced EGFR internalization on clathrin. HSC3 cells were co-transfected with siCYLD-UTR and CYLD deletion mutants. After incubation for 48 h, cells were starved in serum-free medium for 12 h followed by 100 μg/ml CTX treatment for 15 min. CPZ (5 μM) was added before CTX addition. Cell-surface EGFR was analyzed by means of flow cytometry. (C) Effects of full-length wild-type-CYLD and pDEST-HA-CYLD-C601A on cell viability after CTX treatment. HSC3 cells were co-transfected with siCYLD-UTR and CYLD deletion mutants. After incubation for 48 h, cells were starved in serum-free medium for 12 h, followed by addition of 100 μg/ml CTX. After 12 h, cells were harvested and analyzed by using Annexin V-APC and 7-AAD. *$p < 0.005$.

Figure S5

(A)

CYLD expression score	Patients (%)
0	9 (31)
1	7 (24)
2	9 (31)
3	4 (14)

Membrane EGFR score	Patients (%)
0	1 (3)
1	7 (24)
2	10 (34)
3	5 (17)
4	6 (21)

(B)

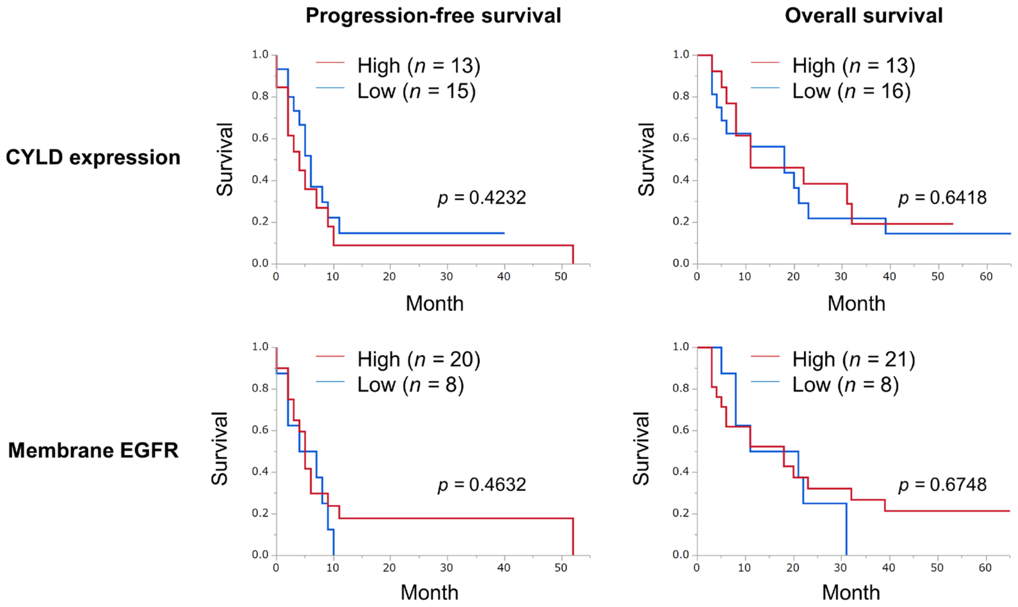

Figure S5. Expression of CYLD and EGFR in primary HNSCC tissues.
(A) Expression scores of EGFR and CYLD in human primary HNSCC tissues as analyzed by means of immunohistochemistry. (B) Kaplan-Meier plots of progression-free survival and overall survival of HNSCC patients with tumors showing low or high CYLD expression and high or low membrane EGFR scores.

Figure S6

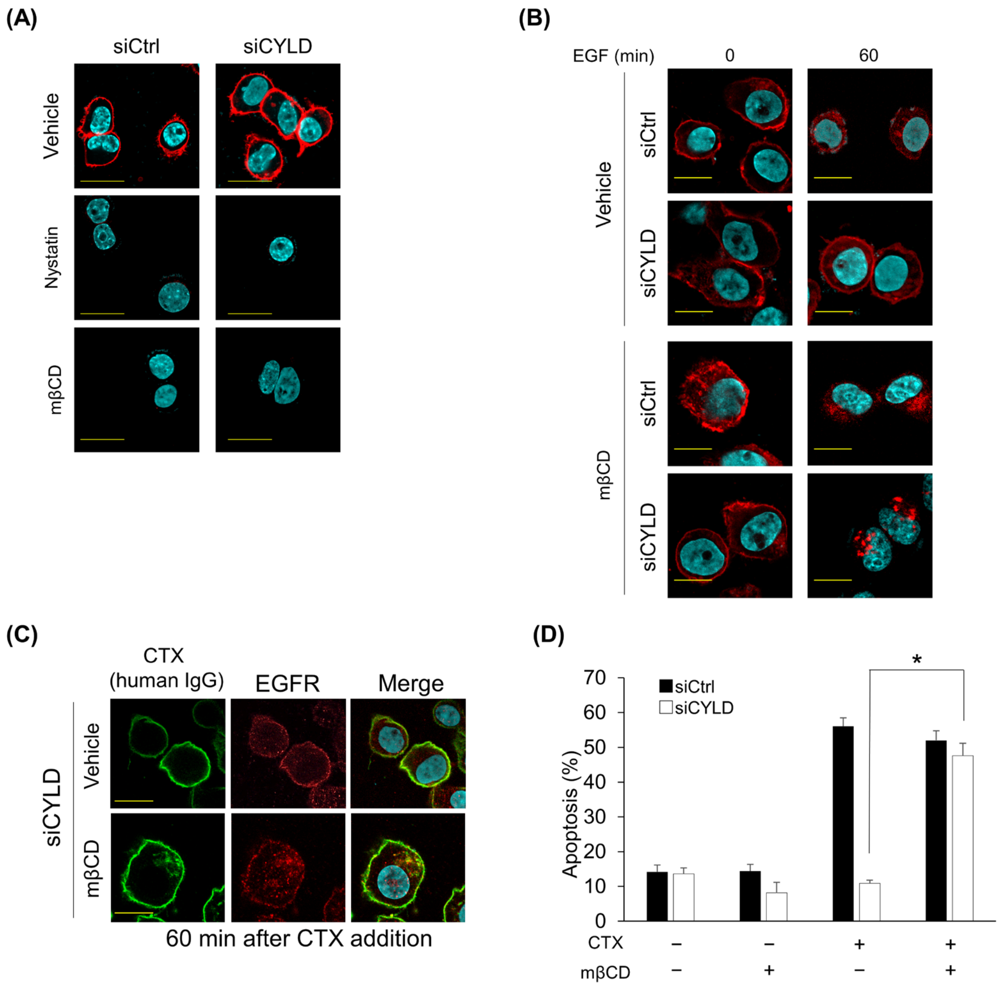

Figure S6. Effects of mβCD on EGFR trafficking and CTX-induced apoptosis in CYLD-down-regulated cells.

(A) Effects of nystatin or mβCD on lipid rafts. HSC3 cells were treated with 25 μg/ml nystatin or 10 mM mβCD for 30 min. Localization of lipid rafts was analyzed by using immunofluorescence staining. Scale bars, 20 μm. (B-C) Effects of mβCD on EGF- or CTX-induced EGFR endocytosis. HSC3 cells were transfected with siRNA and incubated for 48 h. After incubation for 12 h in serum-free medium, cells were pretreated with 10 mM mβCD for 30 min before stimulation with 100 ng/ml EGF for 60 min (B) or 100 μg/ml CTX for 60 min (C). Localization of EGFR and CTX was analyzed by using immunofluorescence staining. Scale bars, 10 μm. (D) Effects of mβCD on CTX-induced apoptosis. HSC3 cells were transfected with siRNA and then incubated for 48 h. Cells were pretreated with 10 mM mβCD for 30 min before treatment with 100 μg/ml CTX in serum-free medium for 12 h. Apoptosis was analyzed by Annexin V-APC and 7-AAD. *$p < 0.005$

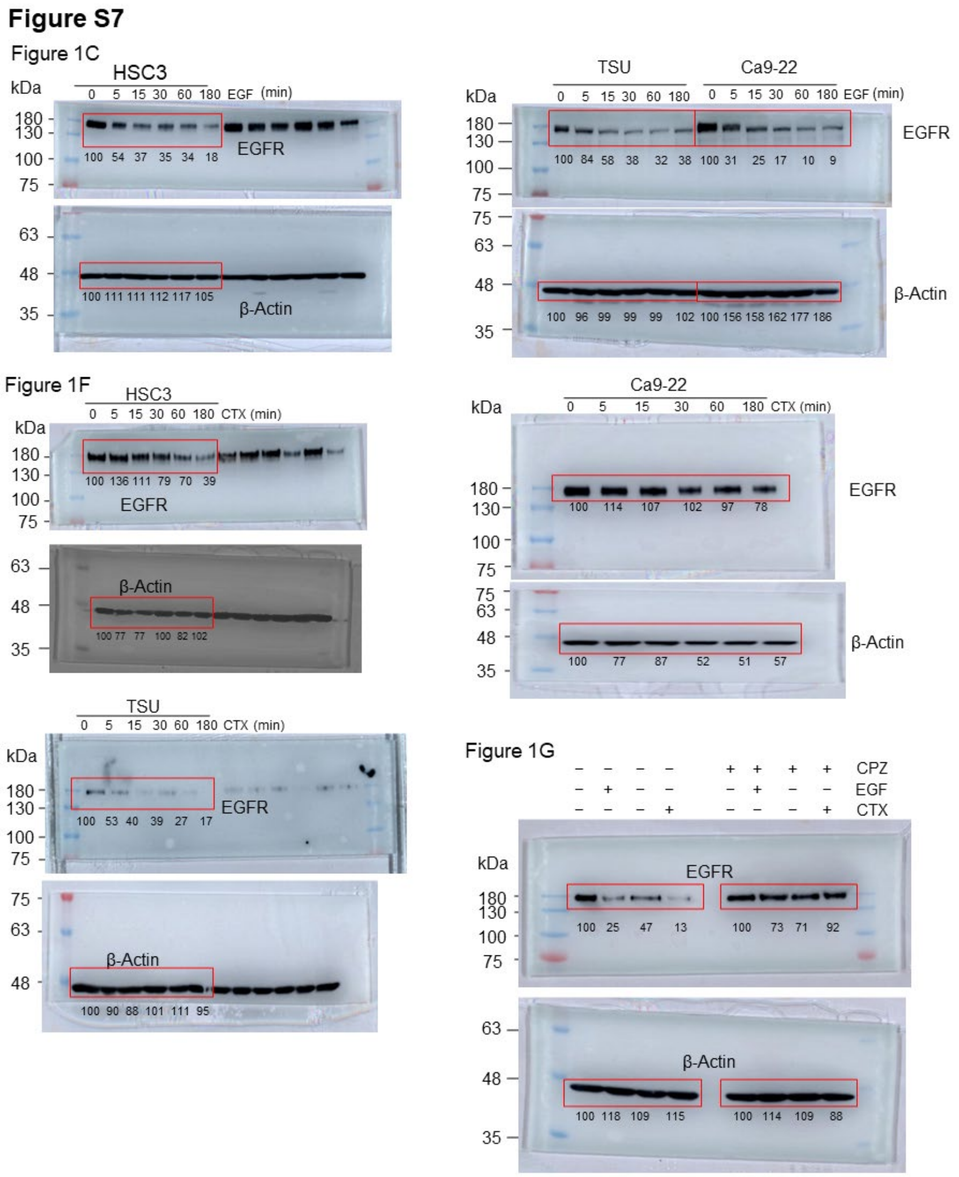

Figure S7. Whole blots for western blots in Figure 1.

Uncropped full-length pictures of western blotting membranes presented in the main Figure 1C, F and G. Some membranes were cut to allow detection by multiple antibodies. Values below the corresponding blot represent densitometric analysis. The values of the proteins other than β-actin were normalized to that of β-actin in the same samples.

Figure S8

Figure 1I

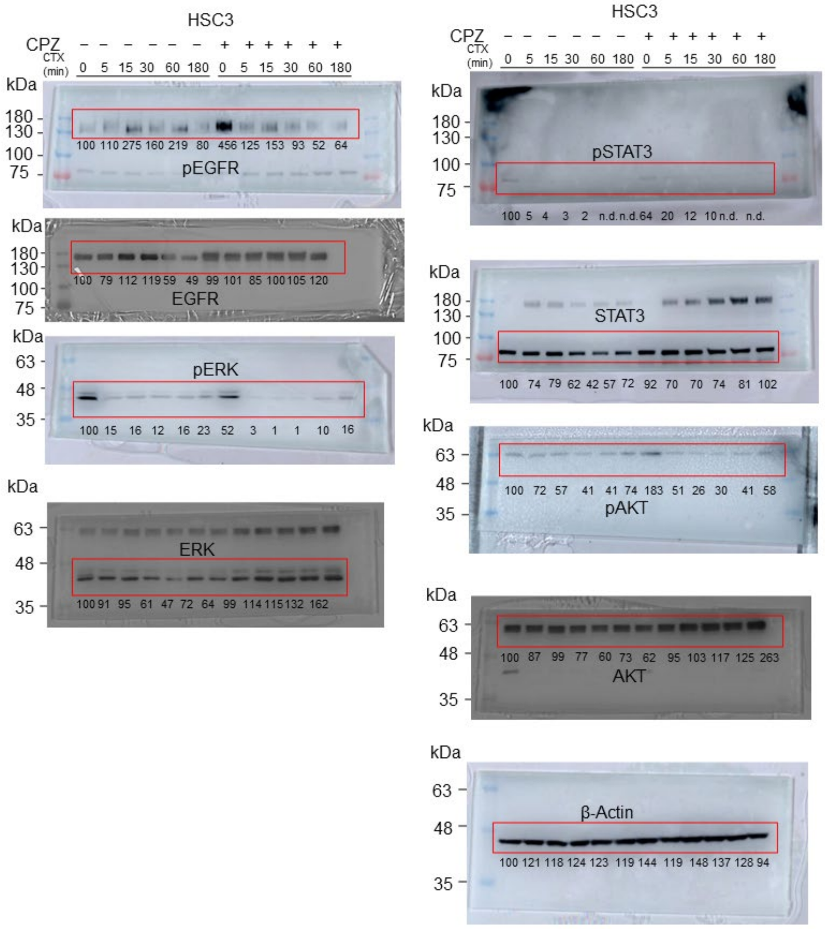

Figure S8. Whole blots for western blots in Figure 1.

Uncropped full-length pictures of western blotting membranes presented in the main Figure1I. Some membranes were cut to allow detection by multiple antibodies. Values below the corresponding blot represent densitometric analysis. The values of the proteins other than β-actin were normalized to that of β-actin in the same samples. n.d.: not detected.

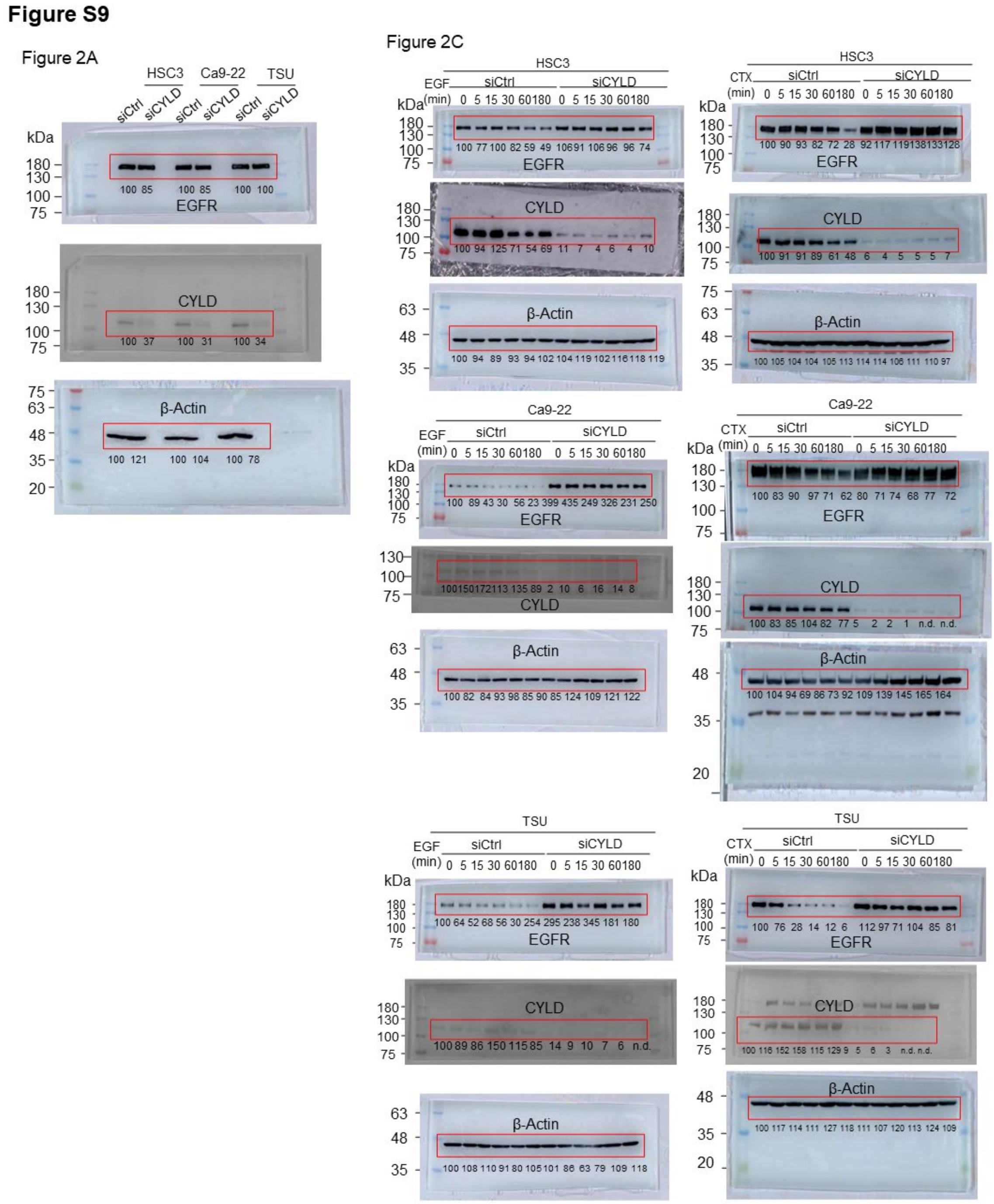

Figure S9. Whole blots for western blots in Figure 2.

Uncropped full-length pictures of western blotting membranes presented in the main Figure 2A and 2C. Some membranes were cut to allow detection by multiple antibodies. Values below the corresponding blot represent densitometric analysis. The values of the proteins other than β-actin were normalized to that of β-actin in the same samples. n.d.: not detected.

Figure 10

Figure 2G

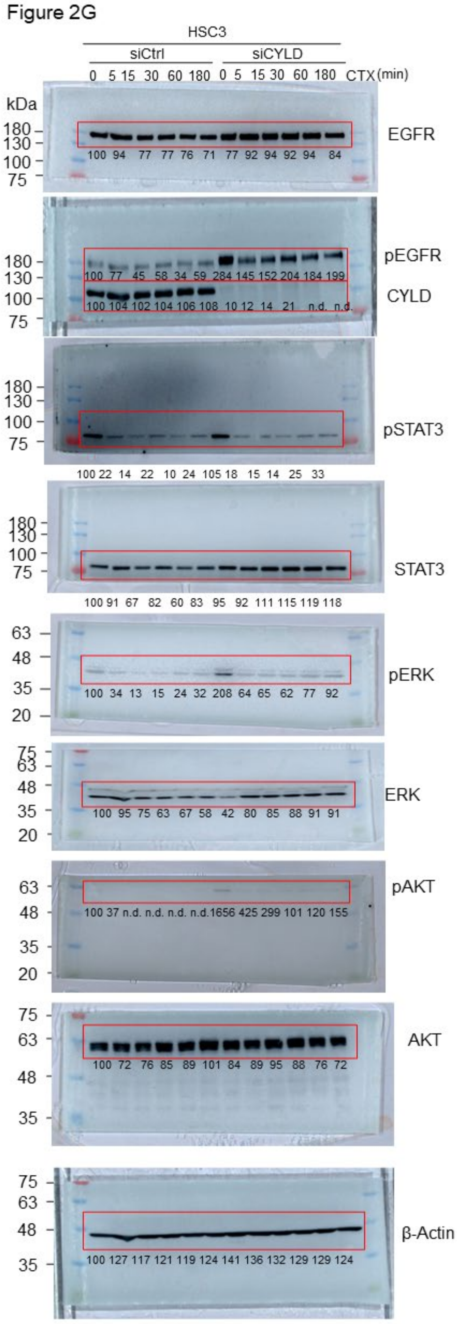

Figure S3B

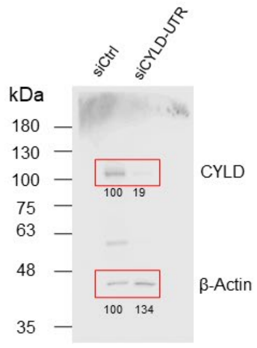

Figure S10. Whole blots for western blots in Figure 2 and Figure S3.

Uncropped full-length pictures of western blotting membranes presented in the main Figure 2G. Some membranes were cut to allow detection by multiple antibodies. Values below the corresponding blot represent densitometric analysis. The values of the proteins other than β-actin were normalized to that of β-actin in the same samples. n.d.: not detected.

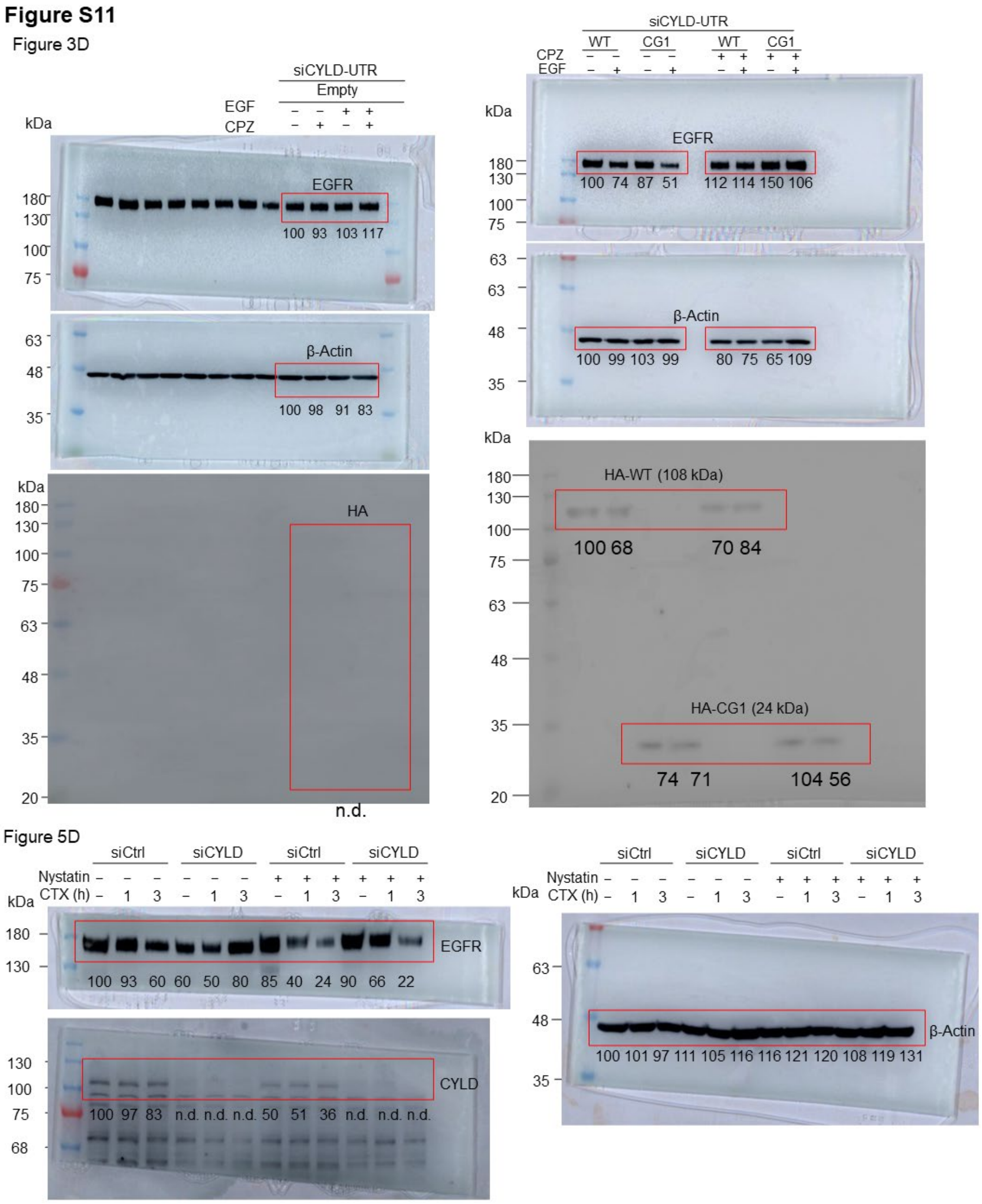

Figure S11. Whole blots for western blots in Figure 3 and 5.

Uncropped full-length pictures of western blotting membranes presented in the main Figure 3D and 5D. Some membranes were cut to allow detection by multiple antibodies. Values below the corresponding blot represent densitometric analysis. The values of the proteins other than β-actin were normalized to that of β-actin in the same samples. n.d.: not detected.
